# Is there a relationship between foot pain and severity of deformity in hallux valgus?

**DOI:** 10.1186/1757-1146-4-S1-O34

**Published:** 2011-05-20

**Authors:** Sheree Nix, Bill Vicenzino, Michelle Smith

**Affiliations:** 1 School of Health and Rehabilitation Sciences (Physiotherapy), The University of Queensland, St Lucia, Brisbane, QLD, 4075, Australia

## Background

Hallux valgus (HV) is a progressive foot deformity involving lateral deviation of the hallux, and a common indication for forefoot surgery. However, the degree of foot pain experienced by individuals with HV is unclear. Recent evidence suggests an association between HV and foot pain; whereas, other studies have found no association. Furthermore, it is unknown whether more severe HV with greater angulation of the hallux is related to more severe foot pain. The aim of this study was to investigate the relationship between foot pain and severity of deformity in healthy adults with HV.

## Methods

Sixty healthy volunteers with HV were recruited (7 men, 53 women; mean age 51.5, range 20 to 75 years). Worst and average foot pain over the past month were determined using Visual Analogue Scales (VAS). Severity of HV was measured as the angle between the hallux and first metatarsal (HV angle) on weight-bearing anteroposterior foot x-rays. Other factors investigated included: body mass index, physical activity, foot posture index, foot mobility magnitude, ankle and first metatarsophalangeal joint ranges of motion, and footwear heel height. Preliminary analyses undertaken have been Pearson’s correlations (p < 0.05). Ongoing analysis will be performed using multiple linear regression modelling to investigate to what extent variation in foot pain may be explained by subject characteristics or structural factors.

## Results

Preliminary analysis (n=30) has shown no linear correlation between HV angle and foot pain (VAS average pain r=-0.20 to 0.08, p>0.05; VAS worst pain r=-0.29 to -0.13, p>0.05). There was a low positive correlation found between average foot pain (VAS) and ankle range of motion (right foot r=0.35, p=0.06; left foot r=0.44, p=0.02). No other pairs of variables were found to be significantly correlated.

## Conclusions

From preliminary analyses, foot pain in individuals with HV does not appear to be determined by the severity of angular deformity. Further analysis will investigate possible combinations of structural factors and subject characteristics that may explain some variation in foot pain related to HV. It is likely that foot pain in individuals with HV is a complex matter influenced by multiple factors that warrant further investigation.

